# SynVectorDB: embedding-based retrieval system for synthetic biology parts

**DOI:** 10.1093/database/baaf088

**Published:** 2026-01-15

**Authors:** Hao Li, Jiani Hu, Jie Song, Wei Zhou

**Affiliations:** Department of Endodontics, Shanghai Ninth People’s Hospital, Shanghai Jiao Tong University School of Medicine, College of Stomatology, Shanghai Jiao Tong University, Shanghai, 200011, China; National Center for Stomatology, National Clinical Research Center for Oral Diseases, Shanghai Key Laboratory of Stomatology, Shanghai, 200025, China; Research and Development Department, Beijing Xunzhu Biotechnology Co. Ltd., Beijing, 100080, China; School of Chemistry and Molecular Biosciences, The University of Queensland, Brisbane, QLD, 4072, Australia; Research and Development Department, Beijing Xunzhu Biotechnology Co. Ltd., Beijing, 100080, China; School of Chemistry and Molecular Biosciences, The University of Queensland, Brisbane, QLD, 4072, Australia; Department of Endodontics, Shanghai Ninth People’s Hospital, Shanghai Jiao Tong University School of Medicine, College of Stomatology, Shanghai Jiao Tong University, Shanghai, 200011, China; National Center for Stomatology, National Clinical Research Center for Oral Diseases, Shanghai Key Laboratory of Stomatology, Shanghai, 200025, China

## Abstract

Synthetic biology part discovery faces significant challenges due to inconsistent data organization and limited semantic search capabilities across existing repositories. We developed SynVectorDB, an embedding-based retrieval system that addresses these limitations through methodological innovations in data integration and AI-driven semantic search. Our approach integrates 19 850 biological parts from multiple sources (Addgene, iGEM Registry, laboratory collections), implementing systematic curation protocols that resulted in 7656 parts achieving verified status through literature-based validation and reliability assessment. We introduce a novel three-level hierarchical classification system organizing parts into functionally coherent categories (DNA Elements, RNA Elements, Coding Sequences, and Application Constructs) with detailed subcategorization. The core technical contribution employs BGE-M3 multilingual embeddings within a scalable vector database architecture to enable semantic similarity matching that significantly outperforms keyword-based retrieval methods. Standardized curation workflows enhance data comparability and search accuracy across heterogeneous sources. The dual deployment architecture ensures high performance through cloud services while maintaining open-source accessibility and deployment flexibility. The system maintains SBOL3 compatibility while providing innovative solutions for biological part organization and retrieval. **Database URL**: SynVectorDB is available in multiple deployment modes: web interface (https://svdb.sjtu.bio), local installation and source code (https://github.com/AilurusBio/synbio-parts-db), and MCP server integration for AI assistants (https://www.npmjs.com/package/synvectordb).

## Introduction

The field of synthetic biology has evolved from theoretical designs to practical engineering applications, necessitating reliable, well-documented genetic parts for robust system construction. While several part repositories exist, they often contain heterogeneous data with varying levels of experimental validation, making it challenging to identify suitable parts for engineering applications. Additionally, existing databases frequently lack standardized classification systems and efficient search mechanisms, leading to time-consuming manual curation efforts [1].

### Related work

Previous studies have attempted to address the challenges in synthetic biology databases through various approaches. The Registry of Standard Biological Parts (iGEM) [2] pioneered the standardization of biological parts, establishing a foundation for synthetic biology databases. Building upon this, the BioBricks Foundation [3] developed comprehensive standards for biological part characterization. More recently, the Synthetic Biology Open Language (SBOL) [4,5] has emerged as a standardized language for describing genetic designs.

Beyond community registries, several software platforms focus on standard-compliant registration and sharing of designs. JBEI-ICE provides an open-source biological parts registry and tools for information management and Web of Registries federation [6]. SynBioHub is an SBOL-native design repository supporting standardized submission, search, and programmatic access [7]. Complementary to these, the BioParts portal aggregates multi-source parts discovery atop ICE infrastructure and extends the Web of Registries concept [8]. More recently, the Freegenes project has created an open-source database of synthetic biology parts with emphasis on accessibility and community contribution [9]. Early analysis of the Registry of Standard Biological Parts highlighted challenges in data quality and curation that persist across platforms [10]. These resources emphasize standards-based curation and repository interoperability [11], while our work focuses on unified semantic retrieval, vector similarity, and natural language interaction, complementing the existing platform ecosystem. Importantly, our approach prioritizes production-grade biological parts that have been validated through experimental use, literature evidence, and commercial applications, ensuring enhanced reliability and practical usability for synthetic biology applications compared to purely theoretical or untested designs.

### Research objectives

This study aims to address the limitations of existing systems through a comprehensive approach. First, the focus is on enhancing data quality through systematic curation and standardized documentation based on literature evidence and community validation. The three-level classification system organizes biological parts into four main categories (DNA Elements, RNA Elements, Coding Sequences, and Application Constructs), providing a structured framework for consistent organization and retrieval.

Technical innovation forms the second pillar of this approach. An embedding-based retrieval system has been developed leveraging advanced vector representations and high-performance vector indexing. This approach integrates semantic understanding with biological domain expertise to optimize search relevance and performance.

Finally, accessibility is prioritized through multiple deployment modes and open-source availability. The system is accessible via web interface (https://svdb.sjtu.bio), GitHub repository (https://github.com/AilurusBio/synbio-parts-db), and npm package (synvectordb) for MCP integration, ensuring researchers worldwide can access this resource through their preferred method.

## Materials and methods

### Data model and sources

We model parts in a relational table with fields including ‘uid’, ‘name’, ‘description’, hierarchical ‘type’ levels (L1/L2/L3), source metadata, and sequence-derived metrics (length, GC%). The SynVectorDB dataset contains approximately 19 850 entries from multiple heterogeneous sources.

The primary data sources include Addgene [12], from which 12 383 functional elements were identified and extracted from deposited plasmid constructs with comprehensive peer-review documentation. The iGEM Registry [2] contributes 4322 BioBrick parts as discrete standardized elements with community validation. SnapGene [13] provides 1367 curated sequences from its commercial sequence library with annotation quality verification. Additional sources include laboratory validation collections (1744 parts) with institutional experimental confirmation.

We consolidated these diverse data sources through comprehensive cross-source deduplication and normalization protocols to ensure data consistency across heterogeneous formats and annotation standards.

### Data curation and standardization process

The heterogeneous nature of biological part descriptions across different sources presents significant standardization challenges. Original part descriptions vary substantially in format, terminology, and completeness, ranging from minimal functional annotations to comprehensive experimental characterizations. To address this inconsistency, we employed manual inspection and literature verification combined with language models to restructure all part information into three standardized categories: part descriptions (functional annotations and biological roles), application descriptions (experimental contexts and use cases), and notes (supplementary remarks and metadata).

Based on our three-level hierarchical classification system, all parts undergo systematic reclassification regardless of their original source categorization. This process involves automated mapping algorithms combined with manual curation to ensure accurate assignment to Level 1 categories (DNA Elements, RNA Elements, Coding Sequences, Application Constructs), Level 2 functional subcategories, and Level 3 specific applications. The majority of parts achieve compatibility with SBOL3 format and classification standards through systematic annotation enhancement and standardized ontology mapping.

Verification status assignment leverages multiple validation sources including literature descriptions, experimental characterizations, and laboratory-based verification protocols. Parts receive verified status when supported by peer-reviewed publications, experimental validation data, or institutional confirmation. This multi-evidence approach ensures that verified parts represent production-ready components with demonstrated functionality, enhancing reliability for downstream synthetic biology applications. The complete data processing workflow is illustrated in Supplementary Fig. S1, with detailed statistics provided in Supplementary Methods.

### AI-driven semantic search system

The search system implements advanced AI techniques to provide accurate and efficient semantic search capabilities. The text vectorization process leverages BGE-M3’s multilingual embedding capabilities [14], generating 1024-dimensional vector representations that capture semantic relationships across different languages and biological terminologies. This approach enables cross-lingual part discovery and supports international research collaboration through unified semantic understanding, addressing limitations of traditional keyword-based search in biomedical databases [15].

The system supports both cloud-native and local deployment modes with optimized technology stacks. For cloud deployment, the architecture utilizes Cloudflare Workers + D1 + Vectorize + BGE-M3 embeddings, where embeddings are generated via Cloudflare Workers AI and queried using Vectorize high-performance vector databases [16]. Metadata filtering leverages D1 SQL databases for precise query processing. For local deployment, the system employs DuckDB with excellent AI/ML integration capabilities, supporting LanceDB vector databases with SentenceTransformer models for reproducible development and offline access. The architecture includes a modern MCP (Model Context Protocol) Server [17] implemented as an npm package, providing standardized AI tool integration and enabling seamless interaction with language models and external systems (detailed API specifications in Supplementary Methods).

### Classification system and SBOL3 compatibility

SynVectorDB implements a comprehensive three-level hierarchical classification system designed for systematic organization of synthetic biology parts:

- **DNA Elements**
  Level 2: Regulatory, Structural, Binding
  Level 3: Promoters, Terminators, RBS, UTR, Origins, PolyA, Homology Arms, Binding Sites
- **Coding Sequences**
  Level 2: Reporter, Enzyme, Membrane Proteins, Regulatory Proteins
  Level 3: Fluorescent/Chromogenic/Luminescent Proteins, Processing Enzymes, Channels, Receptors
- **RNA Elements**
  Level 2: Guide RNA, Regulatory RNA, Structural RNA
  Level 3: CRISPR-related, Riboswitches, Aptamers, Scaffold RNA
- **Application Constructs**
  Level 2: Biosafety, Biosynthesis, Biocontrol
  Level 3: Kill Switches, Metabolic Pathways, Biosensors, Control Circuits

For interoperability, the system provides SBOL3-compatible export functionality. Parts can be exported in both JSON-LD and Turtle formats with Sequence Ontology role mapping derived from the three-level classification system. Additionally, parts can be exported in JSON and FASTA formats, providing flexible data access options for different analysis tools and workflows.

### Database architecture

The architecture of SynVectorDB addresses existing limitations through several integrated components. At its core, a reliable relational database provides data storage, while a vector database enables semantic search through high-dimensional vector indexing of biological part descriptions. This approach allows meaningful retrieval based on functional similarity rather than just keyword matching.

The web-based interface transforms how researchers interact with the database. The interface visualizes the hierarchical classification system, allowing intuitive navigation through the four main categories and their subcategories. Multi-dimensional filtering options enable real-time refinement of search results based on part characteristics, validation status, and source repositories. Interactive charts visualize data distributions, while sequence visualization tools provide direct inspection of genetic elements.

**Figure 1.**
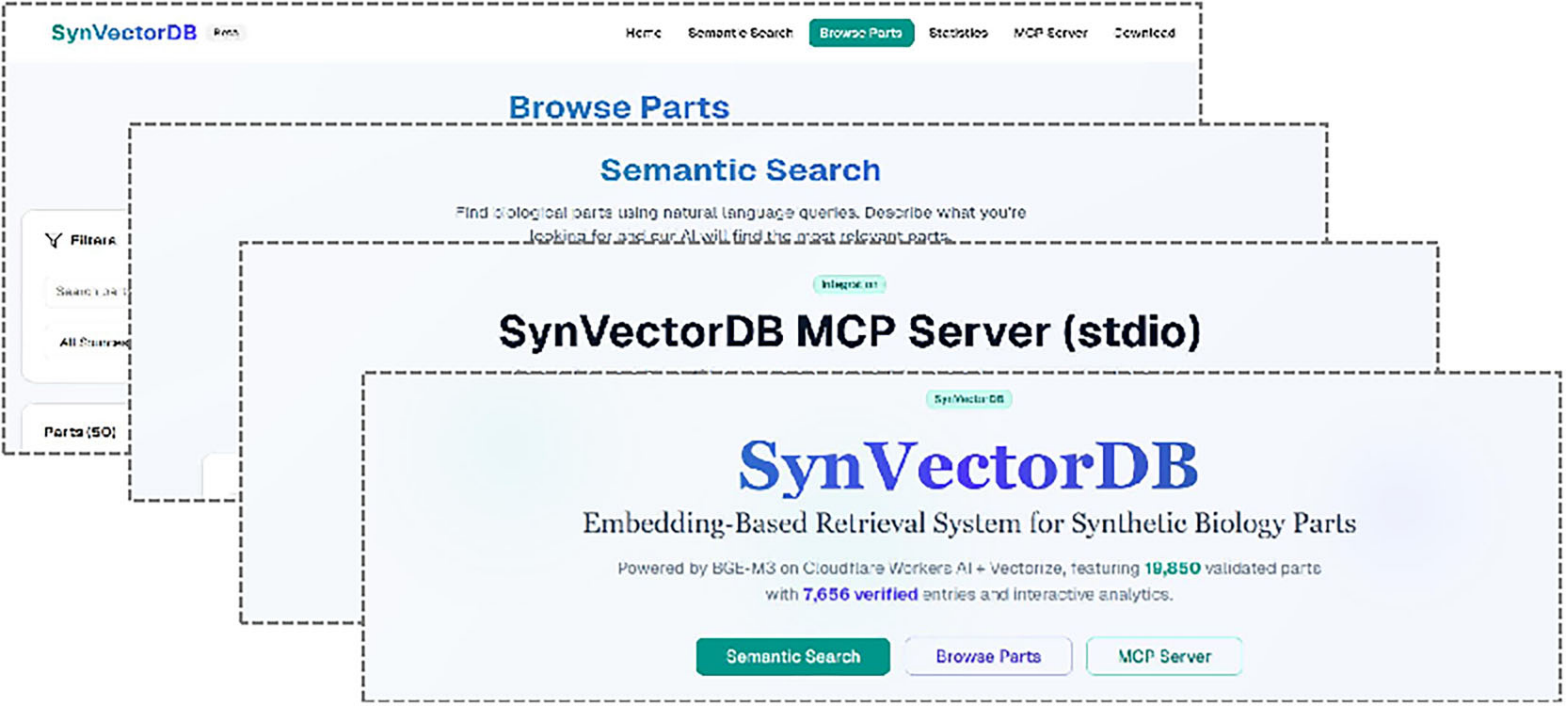
SynVectorDB web interface. The main interface showing the search functionality, database statistics overview, and part discovery features.

Figure 1 demonstrates the main interface of SynVectorDB, showcasing the search functionality, part discovery features, and database overview statistics that enable efficient navigation and exploration of the synthetic biology parts collection.

Behind these user-facing features, the system provides flexible API access with intelligent caching. Batch processing capabilities enable efficient handling of large-scale operations across diverse deployment environments.

**Figure 2.**
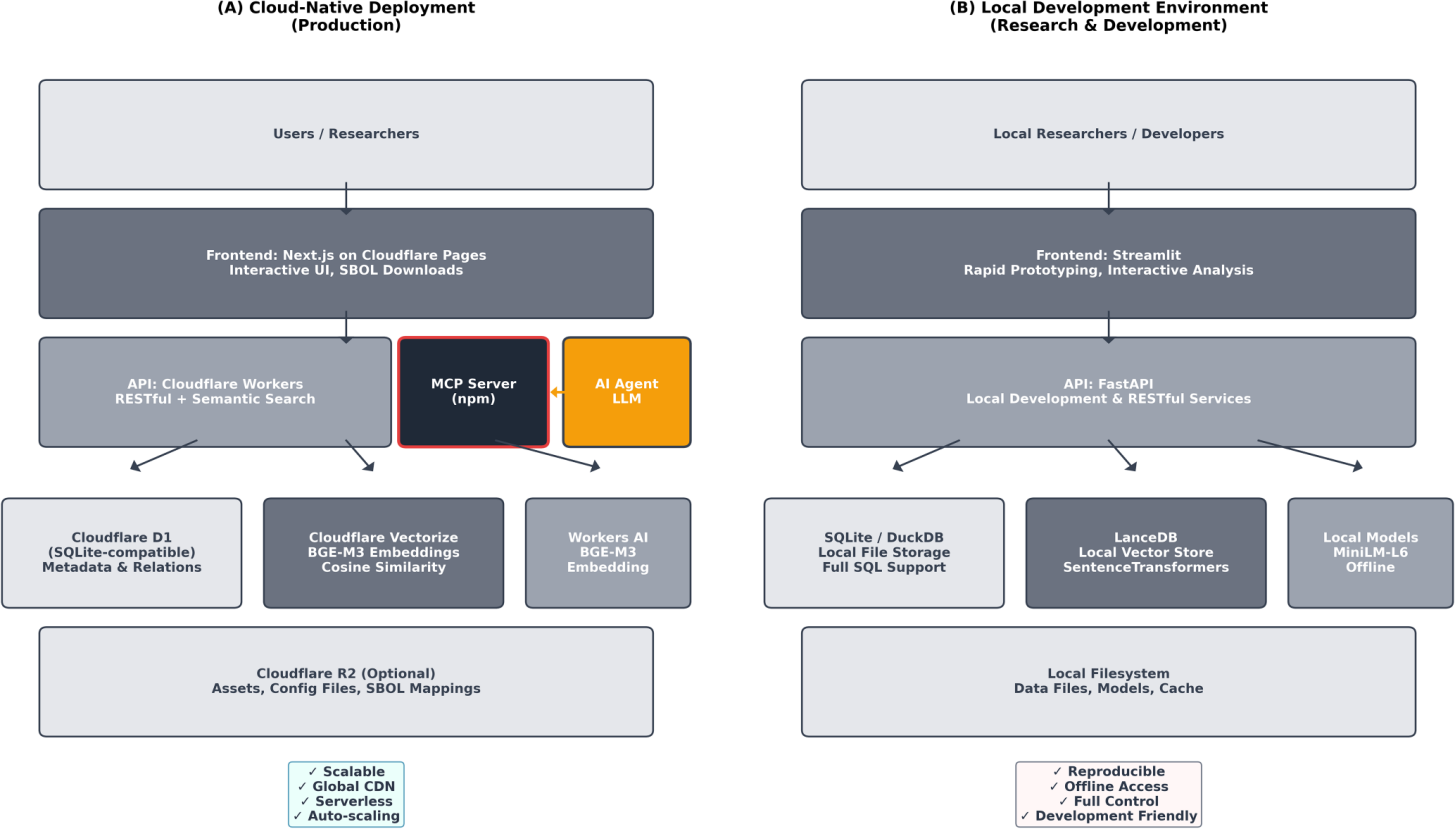
SynVectorDB system architecture. Dual-mode deployment architecture showing (A) cloud-native stack and (B) local development environment.

Figure 2 illustrates the dual-mode deployment architecture, showing both cloud-native and local development configurations.

## Results

### Database content analysis

The database comprises 19 850 parts with comprehensive documentation. Sequence analysis reveals a wide range of lengths, from 1 to 12 461 base pairs, with an average length of 858 base pairs. The length distribution demonstrates the diversity of parts in the database.

Type distribution analysis shows that coding sequences constitute the majority of entries (63.0%, 12 509 entries), followed by DNA elements (33.6%, 6666 entries). RNA elements and application constructs make up smaller proportions (2.7% and 0.7%, respectively). Within these categories, we identified 14 distinct subtypes including membrane proteins, reporters, structural elements, enzymes, and regulatory components.

Source analysis reveals a diverse collection of parts from multiple repositories. Addgene [12] contributes the largest proportion (62.4%, 12 383 parts), followed by the iGEM Registry [2] (21.8%, 4322 parts). Laboratory validation and commercial validation provide significant contributions (8.8% and 6.9%, respectively), while specialized parts from other sources account for the remaining 0.2%. Detailed multi-source data processing workflows and comprehensive source distribution analysis are provided in Supplementary Figs S1 and S2.

Part categorization follows a hierarchical structure with Level 1 categories dominated by Coding Sequences (63%, 12 509 parts) and DNA Elements (33.6%, 6666 parts), with RNA Elements (2.7%, 534 parts) and Application constructs (0.7%, 134 parts) representing specialized functional categories. At the subtype level (Level 2), Reporter proteins lead with 28.1% (5584 parts), followed by Regulatory elements (23%, 4562 parts) and Enzymes (17.9%, 3557 parts), reflecting the predominant focus on characterization and regulatory control in synthetic biology applications. Detailed type distribution analysis including hierarchical categorization, subcategory breakdown, and sequence length distributions is provided in Supplementary Fig. S3.

Verification status analysis shows that 38.6% (7656 parts) have been experimentally verified, while 61.4% (12 194 parts) remain unverified, indicating substantial opportunities for community-driven validation efforts. SBOL3 exports include standardized metadata with Sequence Ontology role annotations and normalized provenance URIs for downstream tool compatibility.

**Figure 3.**
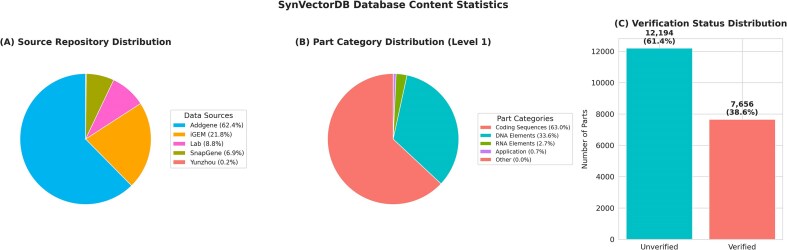
Database content statistics. (A) Source repository distribution, (B) part category distribution (Level 1), and (C) verification status distribution.

Figure 3 provides a comprehensive visualization of the database content statistics, demonstrating the diversity and scope of the curated synthetic biology parts collection.

## Discussion

### Key contributions

The primary contribution of this work is the development of a comprehensive embedding-based retrieval system for curated synthetic biology parts with systematic quality assessment. The focus on data curation and literature-based validation ensures that parts are properly documented with provenance information, providing researchers with reliable metadata for informed selection decisions.

Standardization efforts have resulted in a consistent and reproducible classification system. The hierarchical structure enables efficient organization of parts, while standardized annotations ensure clarity and consistency in descriptions. The addition of SBOL3 export capabilities enhances interoperability with existing synthetic biology tools and workflows.

Accessibility has been prioritized through multiple deployment options. The system supports both cloud-native deployment for public access and local installation for secure, offline use, ensuring researchers worldwide can access this resource according to their specific needs.

### Scalability and architecture considerations

The current architecture utilizes modern database technologies suitable for read-heavy workloads at the present scale. The relational database component handles structured queries and metadata filtering, while the vector database manages high-dimensional similarity searches efficiently.

For future scalability requirements, the system architecture supports several enhancement paths. Higher concurrency scenarios can be addressed through database partitioning and distributed query processing. Performance optimization strategies include intelligent caching layers, batch processing for bulk operations, and background job scheduling for resource-intensive tasks. The modular design ensures that individual components can be scaled independently based on specific performance requirements.

### Limitations and future work

Current records prioritize metadata, sequence, and provenance signals; comprehensive quantitative characterization is not yet included. Future work includes expanding SBOL features (annotated features, constraints, provenance), broadening SO mapping coverage, and enabling community-contributed characterization data.

Detailed source and type distribution analyses are provided in Supplementary Figs S2 and S3, showing the comprehensive coverage across different repositories and biological part categories. The multi-source data processing workflow is illustrated in Supplementary Fig. S1.

## Supplementary Material

baaf088_Supplemental_File

## Data Availability

SynVectorDB is freely available through multiple access points: web interface at https://svdb.sjtu.bio, database download page at https://svdb.sjtu.bio/download, complete source code and documentation on GitHub at https://github.com/AilurusBio/synbio-parts-db, and MCP Server integration via npm package at https://www.npmjs.com/package/synvectordb for AI assistant compatibility. Configure in an MCP Client usage can be referenced in the supplementary documentation. The system supports both cloud-native deployment using Cloudflare infrastructure and local installation with minimum requirements of 4GB RAM and 5GB disk space. The database content is regularly updated with quarterly releases incorporating community contributions and literature updates. Data versioning follows semantic versioning principles, with automated backup procedures ensuring data integrity. API versioning maintains backward compatibility while enabling feature evolution. Community contributions are welcomed through standardized submission protocols via the GitHub issue tracker.
