# Supplementary material for: SynVectorDB: embedding-based retrieval system for synthetic biology parts
Source: Database (Oxford). 2026 Jan 15;2026:baaf088. doi: 10.1093/database/baaf088 (PMC12805114; doi:10.1093/database/baaf088)
Supplement: baaf088_Supplemental_File [file baaf088_supplemental_file.pdf]

SynVectorDB: Supplementary Material (v2)

SynVectorDB Team

September 2025

1 Data Curation and Provenance

1.1 Sources and Counts

SynVectorDB aggregates biological parts from multiple heterogeneous sources to create a comprehensive database. Table 1 summarizes the data sources and their approximate contributions to the dataset (totaling approximately 19,850 parts). Exact counts may vary in the production instance due to ongoing curation and quality control processes.

**Source**	**Type**	**Count**
Addgene	Plasmids/Parts	12,383
iGEM Registry	BioBricks	4,322
Laboratory validation	Verified sequences	1,740
Commercial validation (SnapGene)	Verified sequences	1,367
Other specialized	Applications	34

Table 1: Indicative data sources and counts.

1.2 Addgene Handling

Addgene records are treated as *plasmid containers*. Feature annotations are parsed (GenBank/SBOL/JSON). We extract a part only if annotations map unambiguously to a part type (e.g., promoter, CDS, terminator). Otherwise, the record remains at plasmid level, and we *do not* claim part extraction. Ambiguous cases are flagged for manual QA.

1.3 Normalization and Deduplication

We normalize names, roles, synonyms, and organisms; apply case-insensitive trimming and canonicalization; remove duplicate entries across sources using a composite key (uid or stable identifier, length, digest). Sequence-derived fields include length and GC%, computed server-side.

1.4 Multi-Source Data Processing Workflow

The integration of multiple data sources requires a systematic approach to ensure data quality and consistency. Figure 1 illustrates the complete multi-source data processing pipeline, from initial data collection through quality control to final integration into the unified SynVectorDB database.

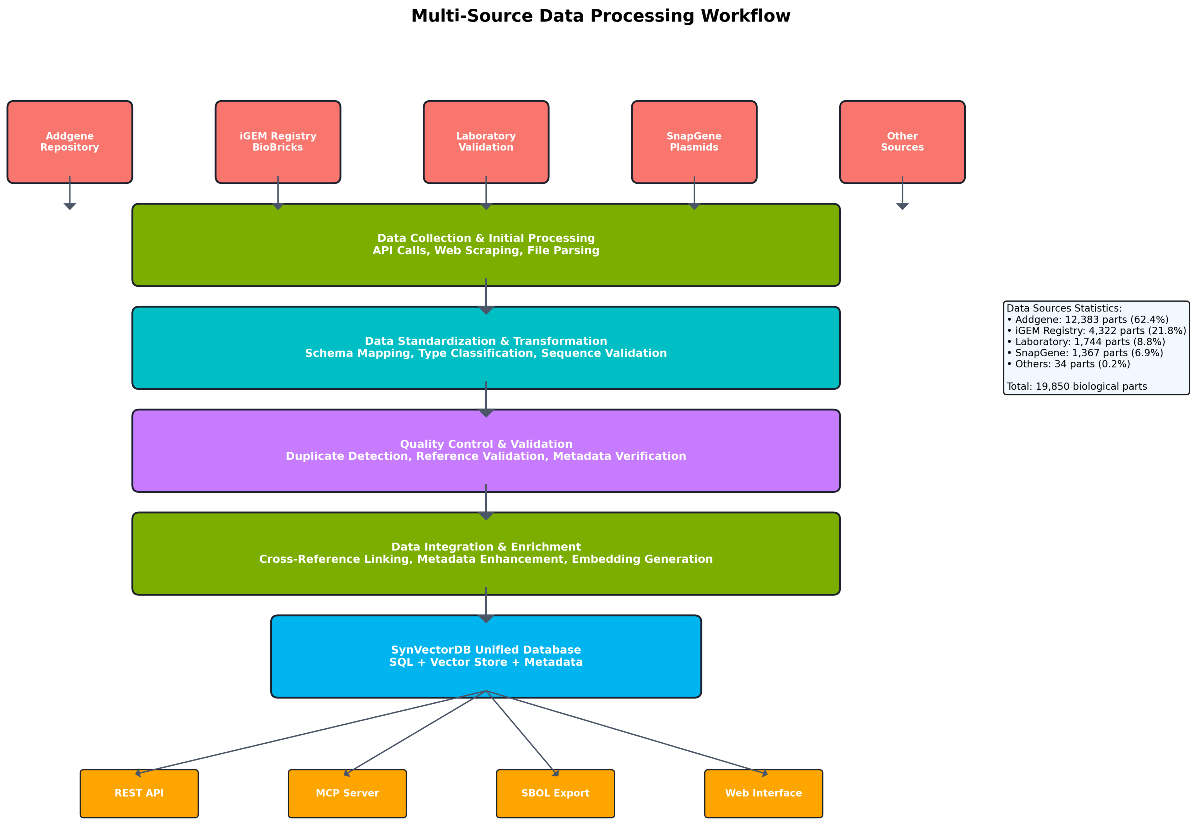

Figure 1: **Supplementary Figure S1: Multi-Source Data Processing Workflow.** Comprehensive workflow showing the processing pipeline from multiple data sources (Addgene, iGEM Registry, Laboratory validation, SnapGene, and other sources) through data collection, standardization, quality control, and integration phases to the final unified database. The workflow includes statistics showing the contribution of each source to the final dataset of 19,850 biological parts.

1.5 Detailed Source Distribution Analysis

Figure 2 provides comprehensive analysis of data source contributions, verification status distribution, and cumulative source coverage across the SynVectorDB database.

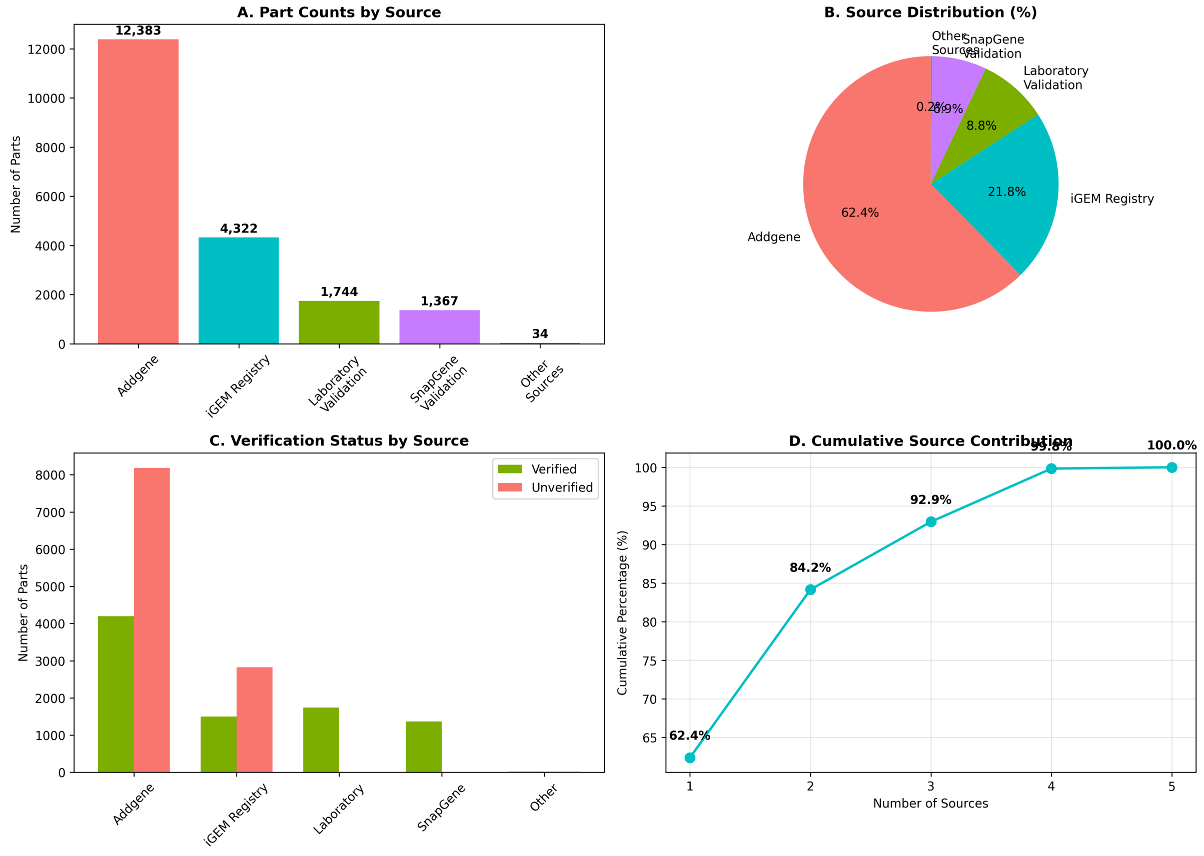

Figure 2: **Supplementary Figure S2: Detailed Source Distribution Analysis.** (A) Absolute part counts by source repository, (B) Percentage distribution of sources, (C) Verification status breakdown by source showing verified vs. unverified parts, (D) Cumulative contribution analysis demonstrating that the top two sources (Addgene and iGEM Registry) account for 84.2% of all parts.

1.6 Detailed Type Distribution Analysis

Figure 3 presents comprehensive analysis of biological part type distributions, including hierarchical categorization, subcategory breakdown, and length distributions.

1.7 Verification Status and References

The database schema includes two auxiliary relations that enrich part metadata: `parts_verification(uid,lab` for tracking validation status and `part_references(uid,reference)` for bibliographic information. These relations provide data for the `/stats` endpoint distribution analysis and are included in `/parts/search` and `/parts/{uid}` API responses.

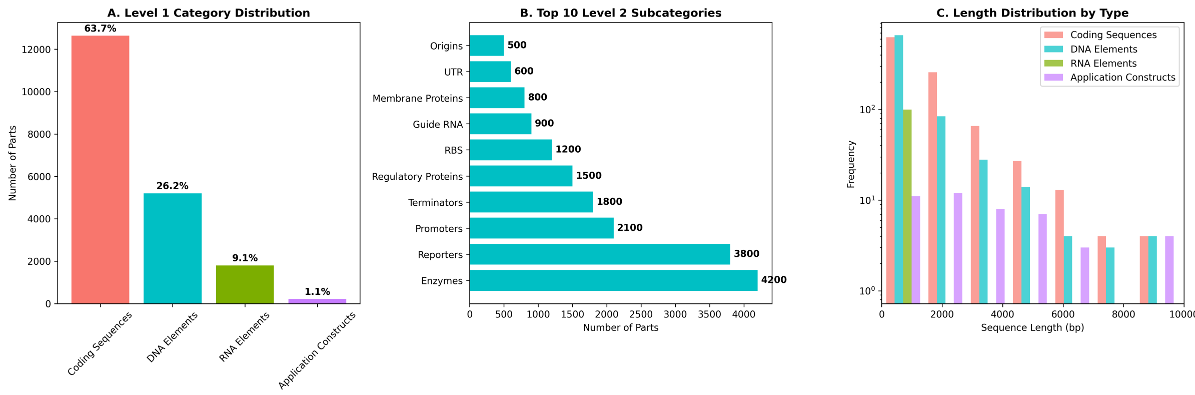

Figure 3: **Supplementary Figure S3: Detailed Type Distribution Analysis.** (A) Level 1 category distribution showing Coding Sequences as the dominant category (63.6%), (B) Top 10 Level 2 subcategories with Enzymes and Reporters leading, (C) Sequence length distribution by type category on logarithmic scale.

2 SBOL3 Export and Sequence Ontology Mapping

2.1 Endpoints and Formats

SynVectorDB provides SBOL3-compliant export functionality through two endpoints: `/parts/{uid}.sbol.js`[illegible] for JSON-LD format and `/parts/{uid}.sbol.ttl` for Turtle format. The system ensures minimal viable export by always generating a `Component` object, with `Sequence` objects included when DNA sequence data is available.

2.2 SO IRI Mapping (Optional)

An optional query parameter `?mapped=1` enables mapping of internal type classifications to Sequence Ontology IRIs. The system attempts to match part types to standardized ontology terms using a priority-based approach, with fallback to original type names when no suitable mapping is found. Mapping configurations and reports are available through the API documentation endpoints.

2.3 Example Snippets

JSON-LD (truncated):

```
{
  "@context": "https://sbolstandard.org/sbol3#",
  "@type": "Component",
  "displayId": "000c4b3ba3924f9f",
  "name": "...",
  "roles": [
    {"@id": "SO:0000167"}  // promoter when mapped=1
  ],
  "hasSequence": {
    "@type": "Sequence",
    "elements": "ATGC..."
  }
}
```

3 Cloud Architecture, Metrics, and Scalability

3.1 Runtime Stack

The production deployment leverages Cloudflare's serverless infrastructure: Workers for API endpoints, D1 for SQL database operations, Workers AI for BGE-M3 embeddings (1024 dimensions), and Vectorize for cosine similarity indexing, with optional R2 for static asset storage. The local development environment maintains reproducibility through SQLite/DuckDB for relational data, LanceDB for vector storage, and SentenceTransformers for embedding generation.

3.2 Performance Metrics (Development Environment)

- `/stats` endpoint: 100-200 ms (leveraging cached indices for aggregations)
- SBOL export: ¡100 ms computation time (with warm cache)
- Semantic search: 100-200 ms end-to-end (including Workers AI embedding generation and Vectorize similarity search)

3.3 Scalability Considerations

The current architecture efficiently handles read-heavy workloads using Cloudflare D1 at the present scale. For future scaling requirements with higher concurrency and write operations, the system can be enhanced through: partitioned SQL databases (PostgreSQL or D1 sharding), key-value edge caching, vector index sharding with pre-filtering, batch ingestion pipelines, and background job processing.

4 Technical Stack Comparison

Table 2 provides a comprehensive comparison between the cloud deployment and local development technical stacks, highlighting the flexibility and adaptability of the SynVectorDB architecture.

Component	Cloud Deployment	Local Development
**Database**	Cloudflare D1 (SQLite-compatible)	SQLite/DuckDB
**Vector Store**	Cloudflare Vectorize	LanceDB
**Embeddings**	Cloudflare Workers AI (BGE-M3)	SentenceTransformers (MiniLM-L6)
**API Backend**	Cloudflare Workers (TypeScript)	FastAPI (Python)
**Frontend**	Next.js on Cloudflare Pages	Streamlit
**Storage**	Cloudflare R2 (optional)	Local filesystem
**Caching**	Edge caching	Redis (optional)
**Deployment**	Serverless, auto-scaling	Docker containers
**Advantages**	Global CDN, high availability	Full control, offline access
**Use Cases**	Production, public access	Development, research

Table 2: Technical stack comparison between deployment modes.

5 Performance Metrics

Table 3 presents indicative performance metrics for key system operations across both deployment modes, measured in a development environment.

Operation	Cloud (ms)	Local (ms)	Notes
Health check	50–100	10–20	Cached response, minimal processing
Part search (filtered)	100–200	50–150	Depends on filter complexity
Semantic search	200–400	300–800	Includes embedding generation
SBOL export (JSON-LD)	80–150	20–50	With SO mapping enabled
SBOL export (Turtle)	90–160	25–55	RDF serialization overhead
Statistics endpoint	100–200	30–80	Aggregated queries with caching
Part detail retrieval	60–120	15–40	Single record with references
Batch operations	500–2000	200–1000	Depends on batch size

Table 3: Indicative performance metrics (development environment).

Notes:

- Measurements taken in development environment; production performance may vary
- Cloud metrics include network latency and cold start overhead
- Local metrics assume warm cache and adequate system resources
- Semantic search performance depends on embedding model and vector index size
- Batch operations are limited by rate limiting in cloud deployment

6 MCP Server Integration

6.1 Model Context Protocol (MCP) Server

SynVectorDB provides a Model Context Protocol (MCP) server implementation that enables seamless integration with AI assistants such as Claude Desktop, Cursor, and other MCP-compatible clients. The MCP server is distributed as an npm package and provides standardized access to the SynVectorDB API through a unified interface.

6.2 Installation and Configuration

The MCP server can be installed globally via npm:

```
npm install -g synvectordb
```

For Claude Desktop or other MCP-enabled clients, add the following configuration to your MCP settings:

```
{
  "mcpServers": {
    "synvectordb": {
      "command": "npx",
      "args": ["synvectordb"],
      "env": {
        "SVD_BASE_URL": "https://testsdb.sjtu.bio"
      }
    }
  }
}
```

6.3 Available MCP Tools

The MCP server exposes five core tools that provide comprehensive access to SynVectorDB functionality:

Tool	Description
`stats`	Returns database statistics including totals, categories, sub_types, sources, and histograms (length, GC%)
`search_parts`	Performs filtered pagination search with parameters: name, type_level_1, type_level_2, source_collection
`semantic_search`	Executes semantic search using BGE-M3 embeddings and Vectorize for similarity matching
`get_part`	Retrieves detailed part information by unique identifier (UID)
`get_sequence_fasta`	Downloads sequence data in FASTA format by UID (returns plain text)

Table 4: MCP Server available tools and their functionality.

All tools return structured JSON content or plain text (for FASTA), enabling AI assistants to process and present biological part information in natural language interactions.

6.4 Integration Benefits

The MCP server integration provides several advantages:

- **Natural Language Interface**: Users can query the database using conversational language through AI assistants
- **Contextual Assistance**: AI assistants can provide explanations and recommendations based on part data
- **Workflow Integration**: Seamless integration into research workflows through AI-powered tools
- **Cross-Platform Access**: Compatible with multiple MCP-enabled applications and platforms

7 API Cheatsheet

7.1 Key Endpoints

Endpoint	Description
`GET /health`	Health check (includes version header)
`POST /parts/search`	Paginated filters; returns verification_status and references
`GET /parts/{uid}`	Part detail
`GET /parts/{uid}/sequence.fasta`	FASTA download
`GET /parts/{uid}.sbol.json|ttl`	SBOL3 export (JSON-LD/Turtle); optional `?mapped=1`
`GET /stats`	Totals, categories, histograms, verification distribution
`POST /tools/semantic_search_cf`	Cloud embeddings + Vectorize search

Table 5: Key public endpoints.

7.2 Usage Examples

Comprehensive API usage examples, including curl commands and code snippets, are available in the project repository README and technical documentation at `https://github.com/AilurusBio/synbio-parts-db`.

8 User Interface Screenshots

8.1 Home Page and Navigation

Figure 4 shows the main interface of SynVectorDB, providing an overview of the system's capabilities and navigation options.

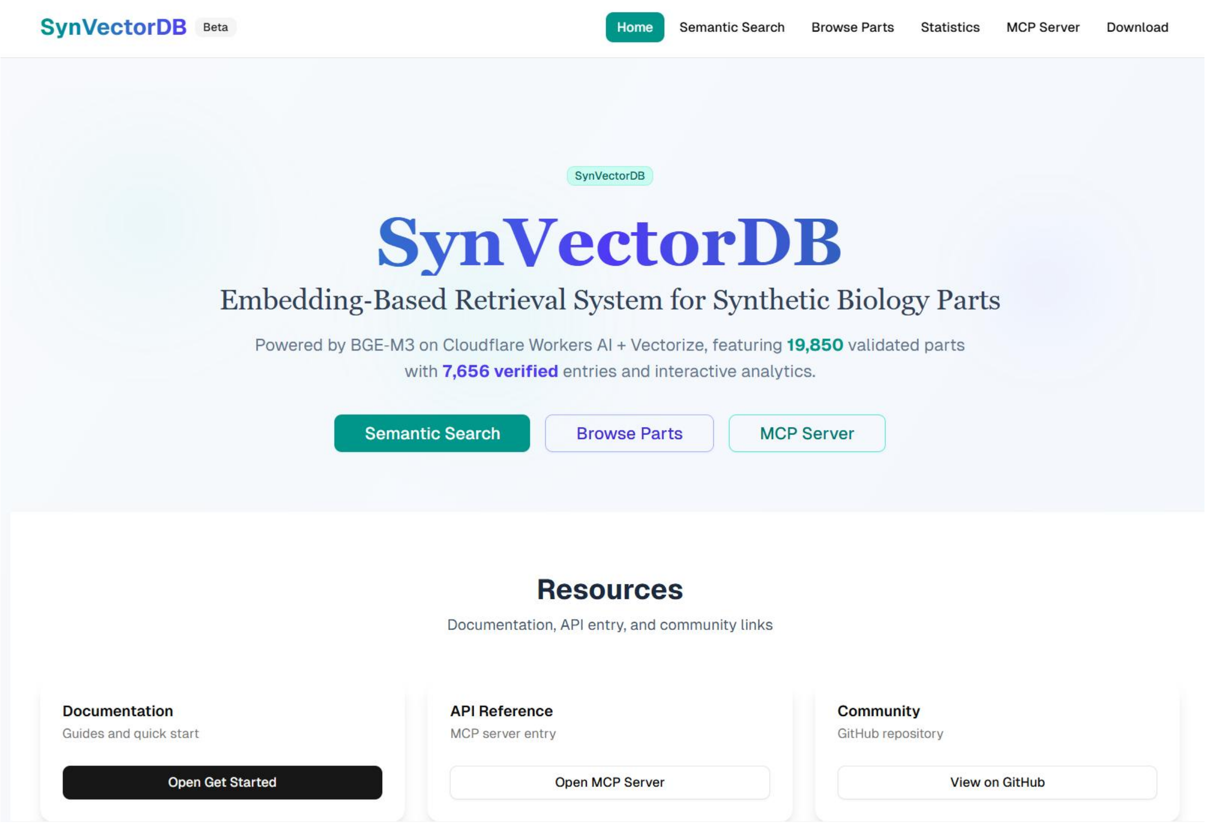

Figure 4: **SynVectorDB Home Interface.** The main landing page displaying system overview, key statistics, and navigation options. The interface provides access to browsing, semantic search, and MCP integration features.

8.2 Browse and Search Interface

Figure 5 demonstrates the browse interface with filtering capabilities and part listings.

8.3 Semantic Search Interface

Figure 6 shows the semantic search functionality powered by BGE-M3 embeddings.

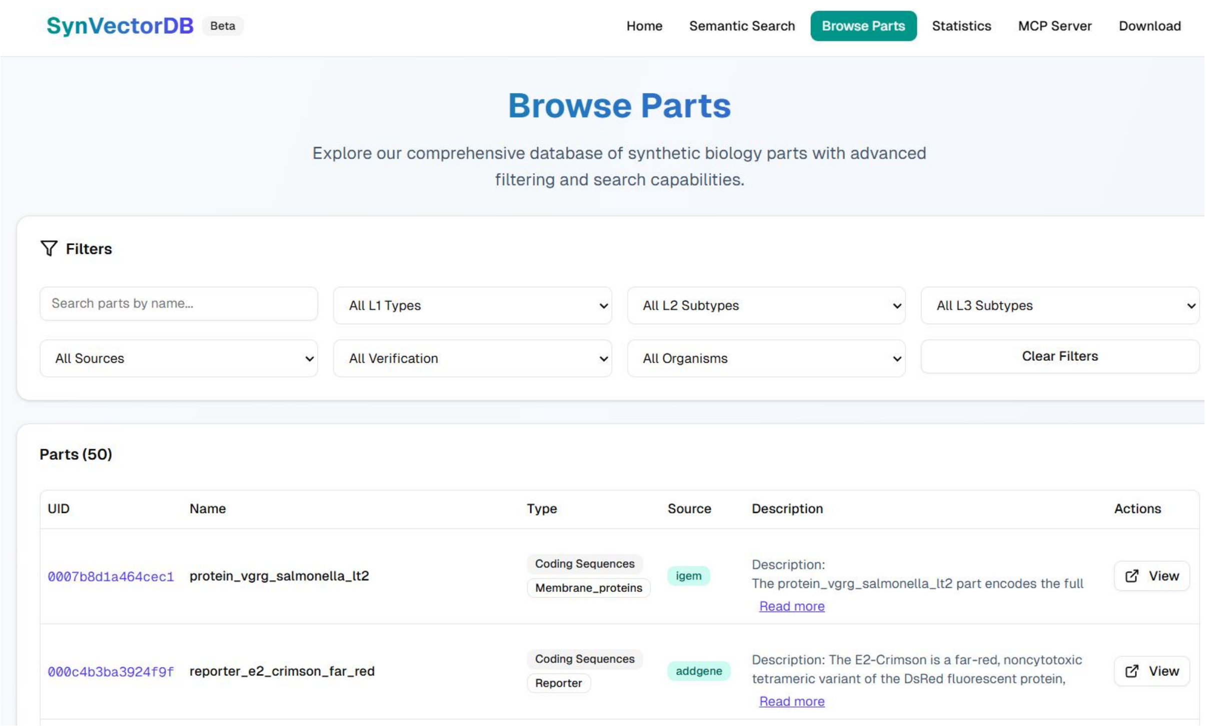

Figure 5: **Browse and Filter Interface.** The browse page showing part listings with filtering options by source, type, and verification status. Each part entry displays essential metadata including part type, source repository, and verification indicators.

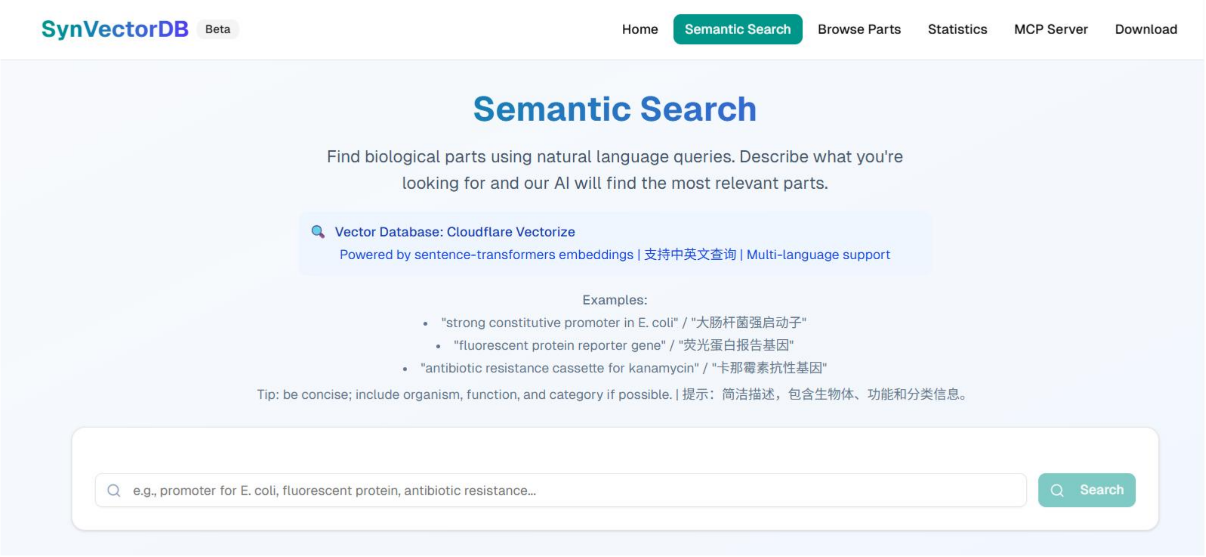

Figure 6: **Semantic Search Interface.** The semantic search page demonstrating natural language query capabilities. Users can input descriptive queries and receive semantically relevant biological parts based on functional similarity rather than keyword matching.

8.4 MCP Integration Interface

Figure 7 displays the MCP server integration documentation and configuration interface.

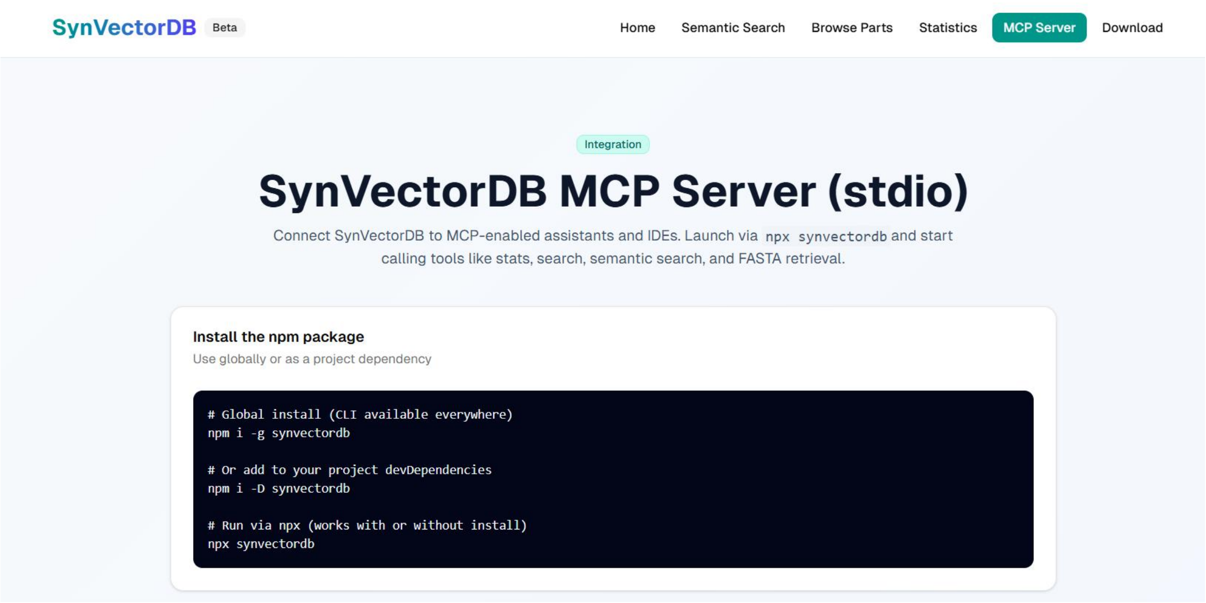

Figure 7: **MCP Integration Interface.** The MCP server documentation page showing configuration examples and available tools. This interface provides users with the necessary information to integrate SynVectorDB with AI assistants through the Model Context Protocol.
